# Designing for Embodied Proximity

**DOI:** 10.1080/10645578.2024.2399487

**Published:** 2024-10-15

**Authors:** Minna O. Nygren, Rhiannon Thomas Jha, Sara Price

**Affiliations:** University College London, London, UK

**Keywords:** Children, design, embodied learning, museum, science learning

## Abstract

This paper draws on notions of embodied learning to inform exhibit design that fosters children’s meaningful embodied engagement to successfully unveil science ideas. While children’s interaction in the museum is often hands-on and speaks to the physical emphasis that embodiment brings, observation of children’s spontaneous engagement at a museum’s Water Zone revealed opportunities and barriers to engagement with, and access to, science ideas in terms of what we call ‘embodied proximity’ and ‘embodied dislocation’. Drawing on design considerations from these findings a set of purpose-built prototype exhibits were developed and deployed to examine how they supported children’s embodied exploration of science. The findings highlight key design dimensions that support children’s accessing and making meaning about science through fostering embodied proximity: considering palette of embodied features; applying direct multisensorial experience; developing temporal-positional contiguity; and designing opportunities for communicating experiences through the body.

## Introduction

Science for young children is often presented through hands-on interactive experiences in science museums. However, relatively little is known about how seemingly subtle embodied experiences, can shape children’s science exploration and understanding. Recent approaches from embodied learning highlight the nuances of multisensory experiences in supporting learning (Thomas Jha et al., 2020; Macedonia, 2019) and point to the need to better understand the role that subtle and routine, sensorimotor experiences play in shaping the ways in which children perceive, develop, and ‘own’ details about science. Embodied learning theory (e.g., Lindgren & Johnson–Glenberg, 2013; Abrahamson and Lindgren, 2014; Mathayas et al., 2019; Nathan, 2022) foregrounds children’s meaning making as a process which is shaped by their interaction experiences, sensing bodies, and actions, a process which the LEAF framework extends to encompass social engagement in embodied interaction (Danish et al., 2020). In turn, designed environments, such as science exhibits, can provide opportunities for young children to engage in meaningful exploratory sensory experiences of science. While children’s interaction in the museum is often hands-on—and therefore speaks to the physical rather than verbal emphasis that embodiment brings (De Carvalho, 2019) - exhibit design may not effectively exploit meaningful ‘embodied’ engagement to successfully communicate science ideas. While the visual plays an important role in interaction, we argue for a closer examination of the “shifting sensory experiences” of hands-on exploration, and their relationship to making science processes transparent, to better understand the nuances of physical interactions that foster children’s effective sense-making around science through the body. A key question is: How can we design to foster meaningful physical interactions in relation to the science idea? How can we foreground this embodied experience for children in museum exhibit design?

This paper reports two studies that aim to address this. First, we observed children’s spontaneous tangible and action experiences at the Water Zone to identify how these experiences shape meaning making—noting design opportunities and barriers to engaging with science ideas, and the implications of this for embodied engagement. From this we identified design considerations to foster embodied access to science ideas. These informed development of prototype exhibits that aimed to promote meaningful, congruent action and sensory forms of interaction in relation to the science idea. We then examined how these exhibits supported children’s embodied exploration of science by attending to how subtle shifts in sensorial accessibility—in terms of embodied proximity and embodied dislocation to science ideas - shaped children’s attention to, engagement with and interpretation of science. We conclude with exhibit design recommendations that consider embodied proximity to sensory engagement with science ideas to foster more meaningful physical interactions.

## Background

### Embodied learning

The field of embodied learning suggests that the way we engage with the world shapes the way we think (e.g., Barsalou, 2008; Abrahamson and Bakker, 2016). This has relevance for museums, where action is central to interactive elements of exhibits designed for young children. Embodied learning moves beyond a high-level notion of hands-on learning to take account of the wider sensory experience in coming to know, to include a focus on manipulation, action on objects, sensing (tactile) objects and their properties, haptic interaction (with e.g., resistance of pumps), and how these are linked to science processes in salient ways (Macedonia, 2019).

According to embodied learning, the activation of multiple sensorimotor systems creates more stable memory traces and knowledge representations (Johnson-Glenberg et al., 2011), and children particularly benefit when actions they engage in are semantically linked to a science idea (Lindgren et al., 2016). Recent research reveals how specific sequences of actions with objects and during social interaction, the action-upon-action exchanges, can foreground meaningful sensorimotor experiences where science is more directly accessed (e.g., Thomas Jha et al., 2020; Nygren et al., 2023). For example, the design of an exhibit, together with social interaction around that exhibit, can provide children with more opportunity to notice, observe and explore the sensorial qualities that the materiality of the exhibit affords (Danish et al., 2020). An adult may also be able to highlight those aspects of the exhibit to a child, thus affording moments of embodied scaffolding to unfold different aspects of science (Price et al., 2020in press; Nygren, Price & Thomas Jha, 2023). Leder (1990) refers to: “the ‘absent body’, where bodies and related motor abilities disappear from conscious awareness, residing in the ‘background’ of experience. Ignored and silenced, we seclude our bodies into our ‘academic (rational) minds’”. This paper speaks to this by exploring in detail *how* children go about meaning making in science museums, foregrounding this embodied experience, and identifying which aspects of exhibits might be successful at enabling children’s direct and multisensory engagement with science ideas.

### Designing for science learning in the museum

Interactive galleries and exhibits are an essential part of museums aiming to attract children and families. These exhibits aim to communicate a science idea and may take many forms, open-ended/task focused, digital/physical/hybrid, small or large scale. Baker-Ward et al., (1990) found that children’s memory for events was enhanced when they performed the action themselves and the actions were goal-directed and familiar, and to some extent when they observed a familiar individual perform an action. Falk and Dierking (2000) highlight the social in supporting learning, through a framework which depicts museum learning occurring through an interrelationship between personal social and physical contexts. Exhibit design typically aims to achieve this through providing opportunities for children and families to engage with ideas through their own actions. The concept of friction may be experienced through slides with different surface materials (e.g., Klaar and Öhman, 2012), or the notion of gravity experienced through a marble run (e.g., Solis et al., 2017). Each experience gives visitors an opportunity to playfully interact with their family and friends within exhibitions (Solis et al., 2017), often designed to afford multisensorial exploration of science ideas (e.g., Allen, 2004; Levent and Pascual-Leone, 2014; Andre et al., 2017). While Allen (2004) explores the challenges of effective interactive design there remain barriers to children’s interactions successfully communicating science ideas through an exhibit. This paper addresses this by taking an embodied learning lens to analyze the shifts in subtle, routine, sensorimotor experiences and how these shape children’s interaction and communication around science.

## Study 1: Spontaneous experiences at the Water Zone

This study explored young children’s spontaneous action experiences in the Water Zone in a museum gallery, to identify how these experiences shape meaning making—noting the design opportunities and barriers in embodied engagement with science ideas. We attended to the perceptual and sensorimotor resources which the exhibit invited, how these were taken up by the children and their role in supporting children’s development of science ideas.

### The Water Zone

The Water Zone forms part of an interactive early years’ science gallery, designed for children aged 3-6 years to ‘discover science through play’. The exhibit comprises a set of interconnected sections that enable different experiences with water including, a deep trough to elicit and observe air bubbles rising, a flow of water from higher to lower that can float plastic boats, gates to block/limit water flow, water wheels and pumps.

### Research design

Families visiting the museum with a child aged between three and six years were invited to participate. Ethical approval was obtained from UCL ethics committee REC 957. Families were informed that we were interested in how the Water Zone supported family interaction around science. Adults were given an information sheet and consent form, and the researcher used an age-appropriate information sheet to talk through the process with the children and gain their assent to take part. Families were encouraged to interact ‘as naturally as possible’. When they moved away from the exhibit, the child (accompanied by their carer) took part in a researcher led semi-structured interview about their interactions. Interactions and interviews were video recorded, although a busy museum floor posed challenges around audio recording of close interactions between child and adult, video recording to minimize inclusion of non-recruited families, and the mobile nature of children’s interaction. Video data from the 16 recruited children (families) comprised interactions with the water exhibit (ranging from 5 to 38 min) followed by a semi-structured interview. Multimodal transcripts (e.g., Jewitt et al., 2016) were produced for each child’s interaction, focusing on how they used their bodies through action, body positioning, movement, tactile exploration, and visual observation to explore science ideas with objects available at the Water Zone. Transcripts for each interview focused on verbal utterances, bodily movement, and gestural forms of communication, e.g., making shapes with their hands, or demonstrating changing speed and direction of movement to convey what they had experienced. Previous work shows how this approach reveals details of children’s conceptions of their experiences with science (e.g., Thomas et al., 2021). Analysis of this data highlighted specific sensorimotor experiences that were salient to children during their interaction, and issues/barriers that arose in the communication of science ideas (names are pseudonymised for reporting).

### Findings: Study 1

Objects in this exhibition afforded several ways of exploring dimensions of water’s dynamic properties through interaction. Children’s multisensory experiences foregrounded touch and tactile sense experiences, and non-tactile experiences (especially visual). The data presented here demonstrates how different actions that can be performed with an interactive exhibit afford different multimodal orchestrations of embodied engagement in relation to science ideas, in terms of embodied proximity and embodied dislocation. The post-interaction interview data suggests that children who spent time interacting with exhibits affording more embodied proximity to a science idea used richer more detailed descriptions of their experience (bodily and verbally). We present three examples from the data to illustrate different forms of embodied proximity and dislocation, and children’s related communication post-interaction.

#### Example 1. Accessing water through tactile interaction

This example demonstrates how subtle shifts in tactile exploration of water underpin a detailed gestural description about aspects of water flow and how water and objects interact from a science perspective (child’s idea). This speaks to embodied proximity with science in the sense that water dynamics are directly felt through the hand.

Meg explored water flow by placing her palm flat on the base of the water trough, holding her palm still, gazing at her hand whilst the water flowed around it. Slowly and with tiny movements of the fingers, she changed the shape of her hand (Figure 2.1). She then rotated a blue dam - attached to the side of the trough with hinges—to stop the water flow (Figure 2.2). As the water level began to rise, she placed her palm at the bottom of the trough again (Figure 2.3). She gazed at her hand, then picked up a yellow boat from the end of the Water Zone, placed it on the water, and watched it float down (Figure 2.4).

**Figure 1.**
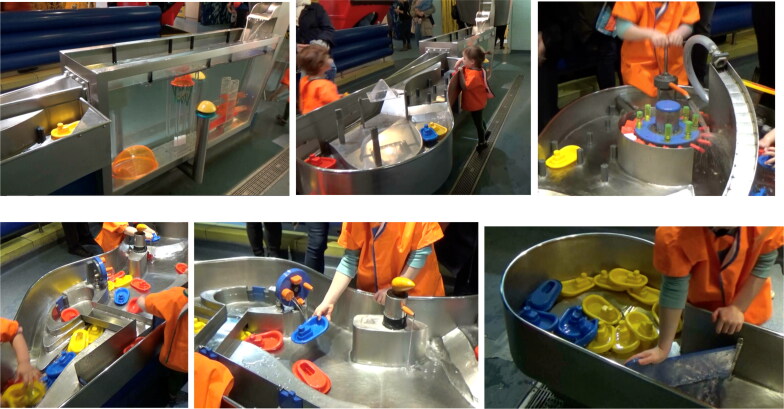
Showing different sections of the Water Zone.

**Figure 2.**
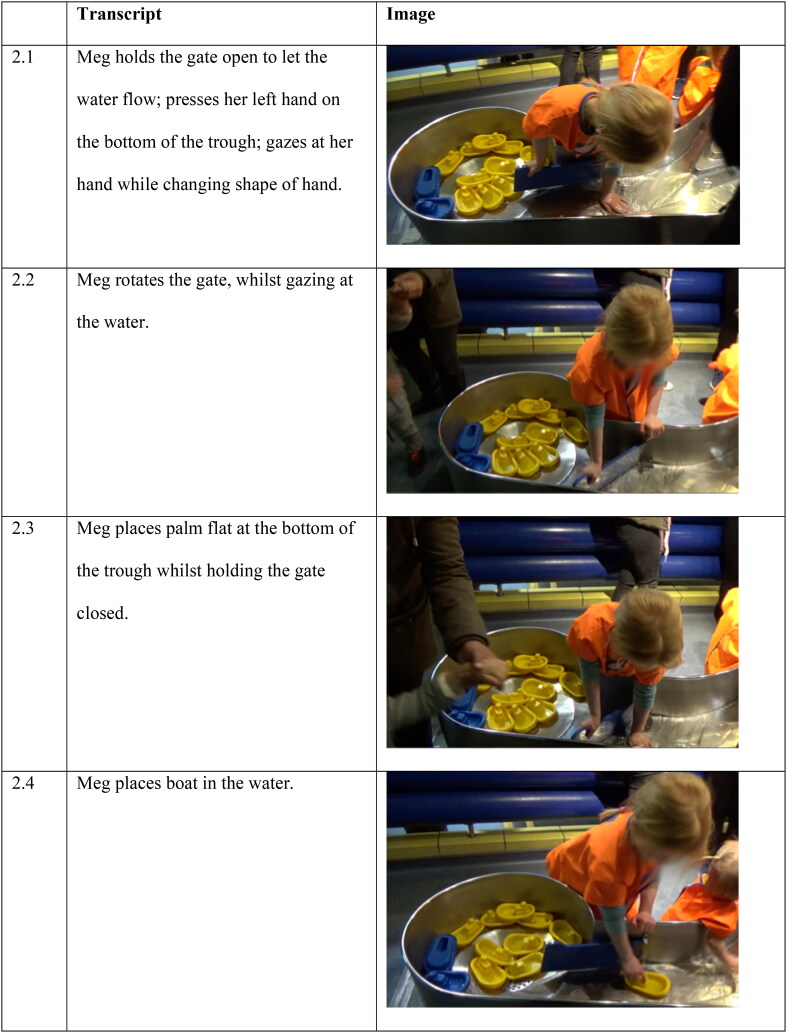
Meg’s interaction with the Water Zone.

**Figure 3.**
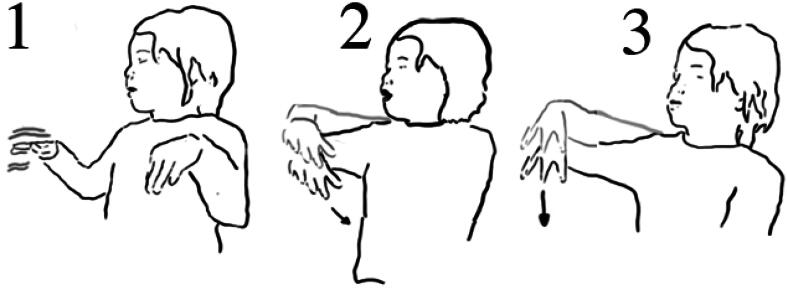
Meg’s gestures (1) how the boats stay above water; (2) the way the boat floated down the waterway; (3) how the water ‘pushed’ the boat downstream.

**Figure 4.**
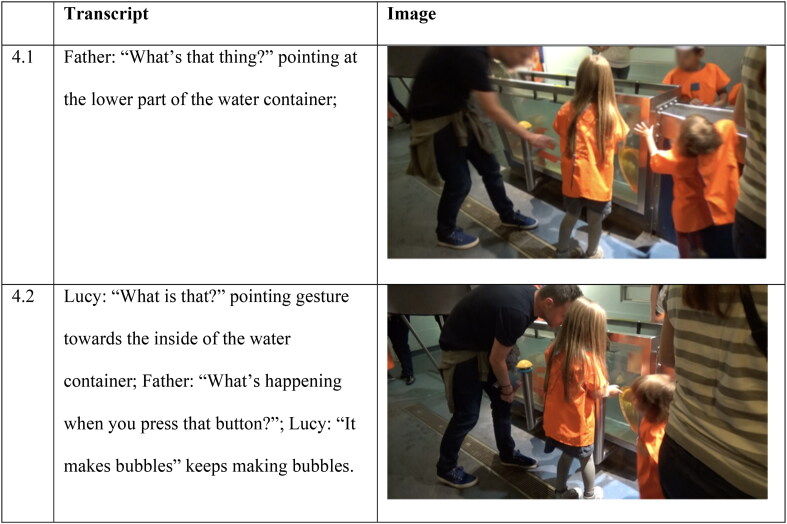
Interaction between Lucy and her father at the Water Zone.

During the researcher-led interview, Meg reenacted dimensions of her experience with water and the objects. Using detailed hand gestures, she demonstrated how the boats stayed on top of the water, how the boats *floated down* the water stream (here, gesture depicting the boats’ movement on the water that was flowing down), and how the water *pushed* the boats *downstream* (Figure 3). The details of these reenactments suggest that the Meg’s sensorial—in this instance, tactile - experience with the water and the objects foregrounded details of *flow of water,* objects *staying on* water, and water *moving* objects.

Meg appears to use her tactile sense as a reflective experience to explore changes in the environment, to see how they change her tactile perception. During her interaction experience she also slowly changed the shape of her hand in the water, enabling a changing sensation of the water flow; and altered other elements, such as opening and closing the dam. This direct access to sensory experience is a form of embodied proximity to science ideas.

#### Example 2. “It makes bubbles”; indirect effect

In contrast, some exhibits, such as the bubble maker at the Water Zone, afforded less opportunity to experience the science process of bubble formation directly through the body. The bubble making machine was triggered by a large yellow button on the outside of a water tank attached to a hollow tube (Figure 4). When the button was pushed in, bubbles formed at the bottom of the water tank, then floated up to the surface. While this is sensorial in the sense that action is required to press a button, the resulting unfolding of the science process is, in effect, ‘dislocated’, both physically (the button pressing action is not sensorially related to bubble making or bubble movement behavior in water) and temporally (the bubbles take time to form enough to have an observable effect).

Lucy and her father are at the top end of the Water Zone. Lucy’s father points to the inside of the water tank, bringing Lucy’s attention to the large yellow button on the outside of the tank (Figure 4.1). When Lucy asks what it is, her father suggests she presses the button. When Lucy presses the button bubbles rise from the bottom of the tank and float to the surface (Figure 4.2). She continues to press the button to make more bubbles and watches them.

**Figure 5.**
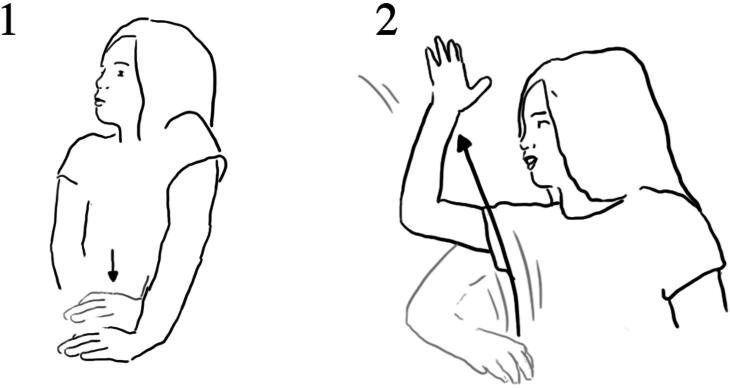
Lucy reenacts pressing the button using both hands (1), then with her right hand moving up she demonstrates how the bubbles moved upwards (2).

In the interview, Lucy showed how she pressed the button, and how the air came out (Figure 5).

Lucy’s communication conveys the process at a relatively superficial level; it focuses on button pressing and the direction of air moving up through the water (Figure 5). While this was an engaging activity, there was less opportunity to build sensorimotor knowledge about how the bubbles were formed, and their movement in water from the action used in the experience. The sensory experience was limited to button pressing, affording little access to sensorial experiencing and knowledge of bubble behavior. While the cause-and-effect is transparent (in terms of ‘push button, bubbles appear’), there is less transparency around how they are formed, where they come from or their relationship to water. We argue that this raises the potential for ‘embodied dislocation’. It raises questions about physical (and digital) buttons that do not map directly to experiencing science. Research within (digital) tangible interaction demonstrates the importance of coherent mappings between schematic action and system response (Price, 2013; Macaranas et al., 2012). Although the bubble exhibit provides a hands-on experience, the action does not necessarily help understand the science idea; the science idea is not embodied in the action.

#### Example 3. Opacity of objects

In this example, interaction with an exhibit is more complex in terms of embodied engagement; it illustrates both embodied proximity and embodied dislocation. Lucy rotates a yellow circular plate that acts as a pump situated at the side of the Water Zone. This is a more sustained action experience (turning a wheel of a pump) where faster exertion generates more water. While Lucy is engaged in rotating the plate, her father (standing behind her) points to where the water is coming out (Figure 6.1).

**Figure 6.**
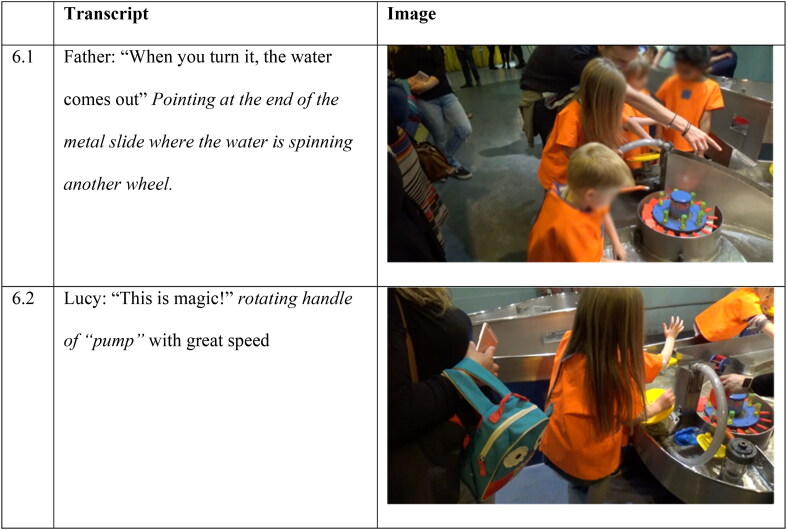
Interaction between father and Lucy at the Water Zone.

Lucy moves away to interact with other parts of the exhibit for nearly five minutes. She then runs back to the plate-pump and rotates the plate again, this time with great speed and force, shouting “This is magic!” (Figure 6.2). Her energetic interaction with the pump, accompanied by the excited exclamation suggests that Lucy was enjoying her experience, yet at the same time, her experience has a dimension of mystery—the underlying mechanism of the pump is hidden - not seen or accessed through her action, i.e., how turning the yellow wheel moves the water so that it exits from the tube. This illustrates both embodied proximity and embodied dislocation. Embodied proximity is present through the tactile and action engagement, where the force one puts into rotating the wheel is proportional to the water running out through the pipe. Embodied dislocation is present given the inability to experience the process of water entering the pump from rotating the wheel and pouring out in another location. The father’s part in this interaction, highlights the role of adult-child interactions in (partially) unveiling the science processes.

##### Interview

**Figure 7.**
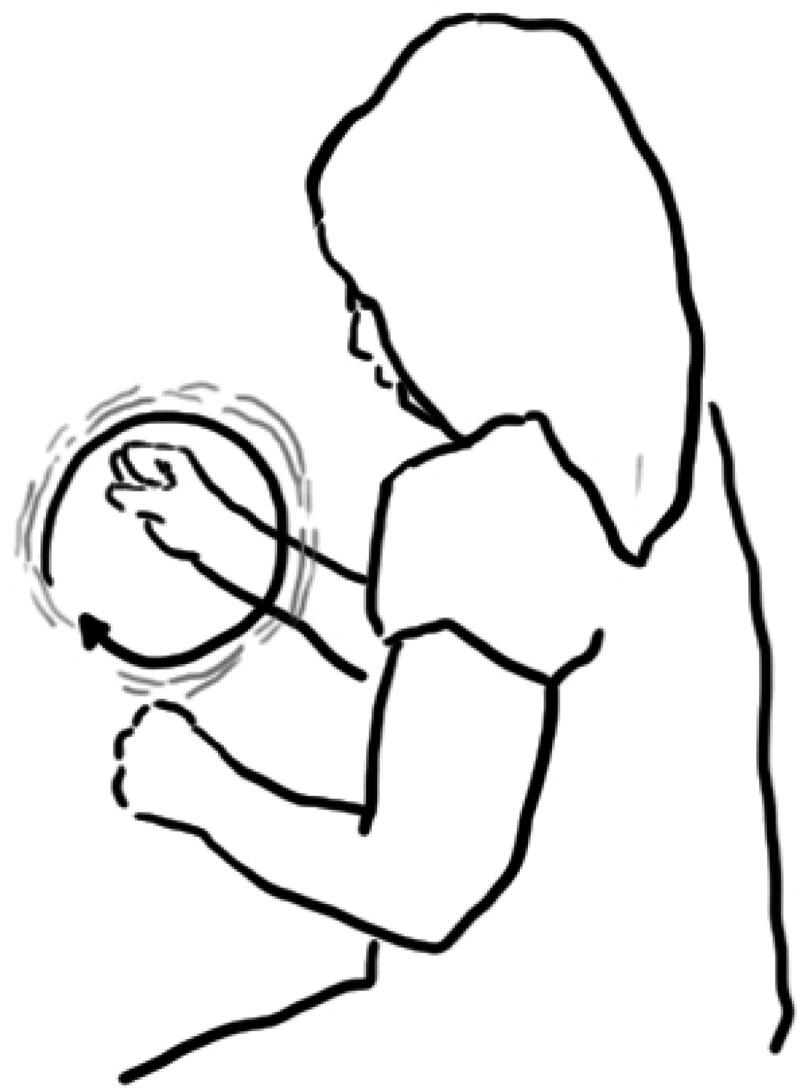
Lucy reenacts the rotating motion of how she engaged with the plate-pump through gesture.

In the interview following Lucy’s interaction, the researcher asked about her exploration with the pump. Lucy responded by reenacting an action of rotating the handle of the pump (Figure 7).

However, she does not elaborate further, verbally or through gesture, about how the water came out or the relationship between her action and the water flow. This may be attributed both to the ‘inner workings’ of the exhibit being obscured (the yellow wheel-plate that is rotated and the metallic part that is attached to the pump do not show how the rotation is connected to the water being picked up) and the physical distance between location of action and location of water exit.

### Design considerations for embodied proximity

The above findings raise questions around embodied proximity and embodied dislocation - or the specific sensory experiences in relation to science ideas and point to design considerations for science exhibits for young children from an embodiment perspective. Experiences where embodied proximity was evident led to children’s communication that involved greater level of detail in relation to the science idea. This raises a question around how exhibit designs that incorporate a notion of embodied proximity might shape children’s later communication around related science ideas. Based on these findings, we devised three high-level design considerations for science exhibits to foster embodied proximity through increasing cause-and-effect transparency: 1) identifying sensorimotor interactive features of an exhibit linked to science ideas; 2) fostering direct sensory interaction that exposes aspects of science ideas; 3) developing temporal-positional contiguity related to action with an exhibit, i.e., how closely positioning and time are linked to a science idea.

In a science exhibit that involves action-input from the visitor, making the action’s effect immediately available can help the child experience the process that their action has started. This can be done through incorporating transparency into design of opaque objects to support access to underlying mechanisms. This may be visual, but we propose the value in also providing other sensorial access including *feeling* or *dynamic touch* that relates to the science process. For example, rather than pressing a button to create air bubbles, some form of ‘blowing’ (e.g., bellows) could create the bubbles, and together with the opportunity to feel how the air goes through the water, could support the visitor’s awareness and understanding. In so doing, the design encourages ‘embodied proximity’ that affords direct sensory experience of science ideas, and where action and effect are co-located or clearly linked.

## Study 2: Exhibit designs from an embodied proximity perspective

### Study design

Three water exhibit prototypes were developed utilizing the design considerations outlined above to explore ways in which embodied proximity might encourage exploration and communication of science ideas (Table 1, 2 and 3). Through focusing on specific attributes of the objects, design choices were made to address gaps in designing for embodied proximity through attention to; sensorimotor features, direct and sensorial experience, developing temporal-positional contiguity. The tables below detail how these elements aimed to foster embodied sensorimotor experiences to effectively support access to science ideas.

#### Materials: designed objects

**Figure 8.**
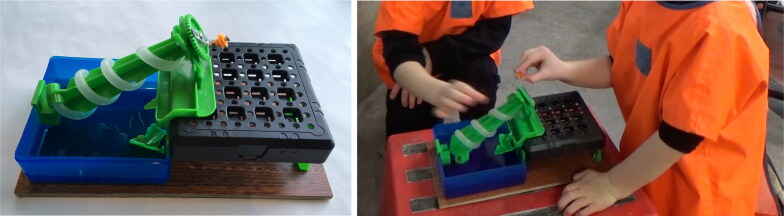
Recycle screw pump (Connex Amazing Toys Ltd).

*Archimedes Screw* was an off-the-shelf recycle screw pump from Connex Amazing Toys Ltd (Figure 8). Turning a small handle clockwise at the base of the object causes water from the blue basin to move upwards through the transparent plastic tube to the top, from where it falls back into the basin. The water was colored using food dye to make the water’s movement more visible.

##### Water wheels

**Figure 9.**
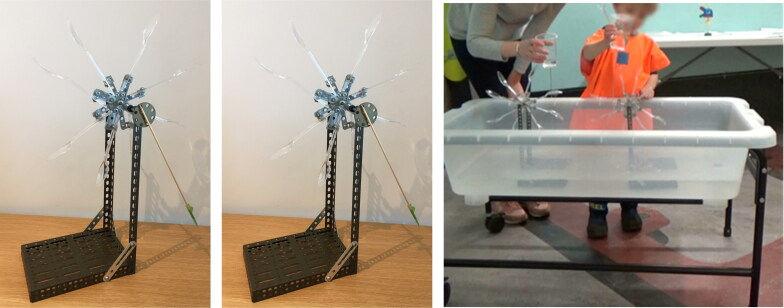
Water wheels, with teaspoons, tablespoons, and in the water table.

Two water wheels (Figure 9) each comprised stainless-steel flat bases (that could rest steadily at the bottom of the water table), with two vertical 20 cm beams holding a rotating wheel at the top, made up of several transparent spoons. The water wheels were identical apart from the size of the blades; one made with teaspoons, the other with tablespoons. Transparent cups were used to pour and create a flow of water; one cup had a hole at the base (filling it with water and holding it up resulted in a slow steady flow of water), another cup had no hole (filling it with water and pouring from the top released more volume of water more quickly). The rotation speed of the wheel changed depending on how much water was poured and the rate at which it was released.

**Figure 10.**
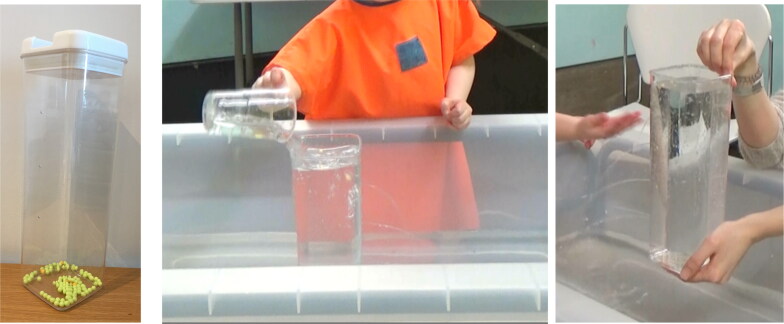
Water tub, and water flowing from holes in the tub.

*Water tub:* was made from a plastic storage jar with four holes vertically spaced up one side of the jar (Figure 10). When the jar was filled with water, water flow trajectories differed from each of the holes. As the water level dropped the flow ceased first from the highest hole, and so on. Colored plastic beads floated on the surface of the water indicating the water level.

While the Archimedes screw was stand alone, the water wheels and water tub were designed to be used within a large translucent rectangular water tub (112 cm x 62 cm) filled with fifty liters of water.

#### Procedure

Nineteen families, with at least one child between 3 and 6 years old, were recruited on a voluntary basis to interact with the purposely designed objects in a water table. To overcome challenges around ethics and video recording experienced in study 1, this study took place in a room adjoining the Water Zone, creating a similar environment to the museum floor. Families were informed that the study aimed to explore how these objects supported family and child interaction to inform future exhibit design. They were encouraged to interact together as naturally as possible, while the researchers provided practical support where necessary. In twelve families the adult was the primary interactant with the child. In the remaining seven, children were left to explore on their own (which is not atypical of the in-gallery interaction space) and the researcher took a more active approach in supporting children’s exploration, where necessary. In these instances, interactions were always child-led. The researcher supported children to interact with the exhibit, and to talk about what they were doing and seeing. Researchers did not offer up any explanations and did not pass judgment about any suggestions that children made. Interactions with the objects ranged from (10-23 min, average of 16), and interviews from (3-15 min, average of 7.6). Interview questions prompted children to talk about their experience of interacting with each object, to describe what they did and how water moved in relation to that object. All interactions and interviews were video recorded, the camera being placed to the side to be as unobtrusive as possible, but to record children’s whole body in the frame. Ethical process was the same as for study 1, and all names are pseudonymised for reporting.

#### Analysis

The analysis began with two researchers watching each video once, making general notes about young children interacting with science-themed objects. On the second viewing, they looked at how children used action to explore science ideas with the objects. On the third viewing they marked down instances where children drew on their experiences at the water table in the post-interaction interviews through verbal description and re-enaction through their bodies. In so doing, the analysis revealed how the design of the objects shaped children’s descriptions of their experiences with science ideas, and what these details could tell us about the relationship between the design of the objects and the child’s access and experience with science from a sensorimotor interaction perspective.

### Findings

All participating children interacted with the three objects, accessing science ideas through their actions. During the post-interaction interview, children reflected on their experience communicating with the researcher. While there were differences in the modality (verbal and/or gestural) and quality of expression (detailed verbal descriptions vs monosyllabic responses, detailed gestural representations vs nondescript gestures), perhaps due to shyness or age, the analysis nevertheless revealed details of how children’s experience with science-themed objects supported their science thinking. Here, we present two examples with each of the objects that speak to the design perspectives adopted in the object prototypes. Illustrative examples are taken from post-interaction interviews, since these demonstrate the details of how children experienced and perceived the dimensions of science they had accessed during their interaction. The findings speak to the notion of embodied proximity and dislocation, in terms of what kinds of interaction and engagement the objects afforded, and the level of detail and depth children could interact, and later communicate, about their science idea.

#### Archimedes screw

Through interaction with the Archimedes screw children were able to make connections between their action and the mechanism of the Archimedes screw. Here we illustrate this with Anna and Elsa, and Ezra and Jude.

##### Anna and Elsa

**Figure 11.**
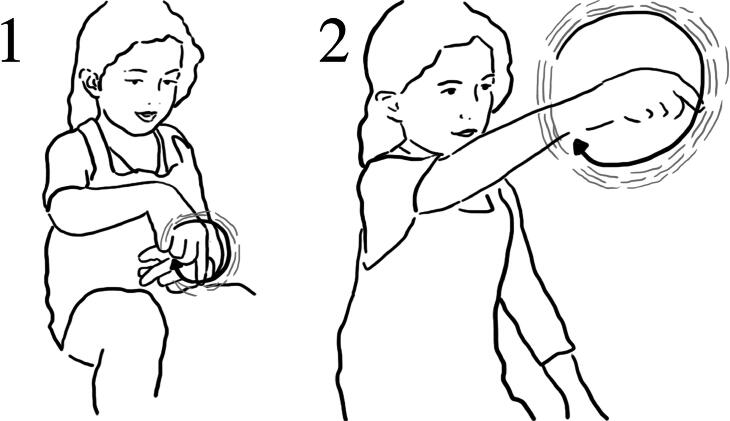
(1) Anna: Well, it came out from this (gesturing a spinning motion); (2) because of the power of how I spin it (makes a large round circular spinning motion in the air with her index finger).

In her post interaction interview Anna talked about her interaction with the Archimedes screw and demonstrated her experience through gesture. In response to the researcher’s question “How did it [the water] come out?”, she verbally explained whilst also gesturing using an upward spiral motion with her hand and finger to demonstrate both her own action with the object and the result this had on the motion of the water (Figures 11(1) and 11(2)).

Through gesture, Anna elaborates her verbal expression about her experience with the Screw, to demonstrate how the water moved through the ‘screw’, by referring to her own rotating action as the initiator of the process. She made a link between her action and its effect on the Screw. We argue that the embodied features, direct multisensorial experience, and temporal-positional contiguity, related to action supported such linking.

**Figure 12.**
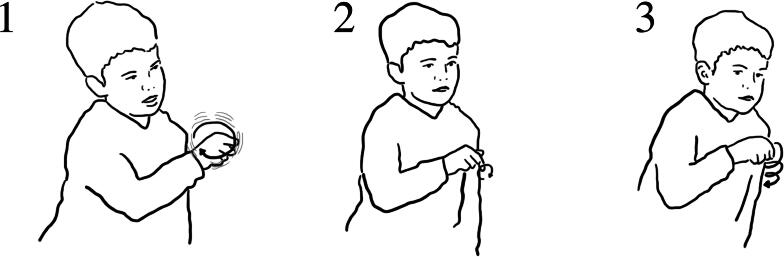
(1) Jude: It, uhm (*gestures a spinning motion in air*); (2) when you turn that bit around…(makes a very small spinning motion in air); (3) pipe (*spinning motion*). It wiggles (*gestures a spinning motion as above*.).

**Figure 13.**
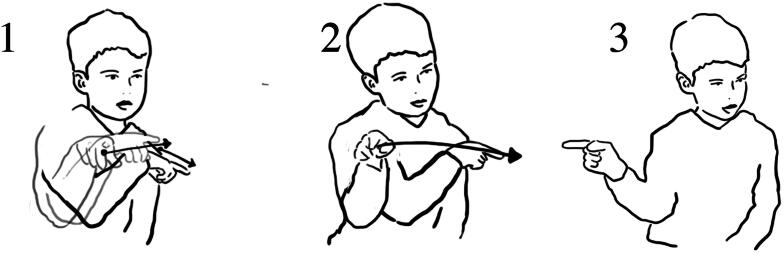
[R1 Why did the water go up?] (1) Jude: Because it went forwards (*moves pointing finger toward his left in small increments*); (2) this way… (*pointing gesture toward his left*); (3) not that way (*pointing gesture toward his right*).

**Figure 14.**
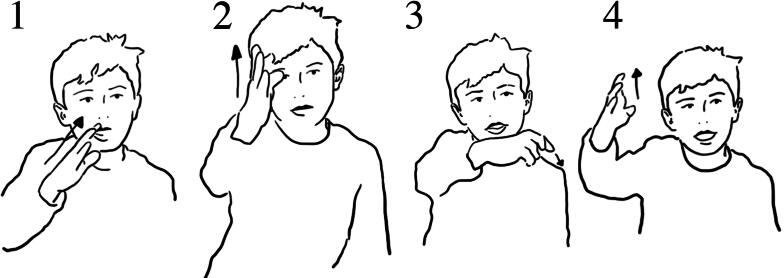
(1) Ezra: “part of it uhm, was going up; (2) then when it went up to the top; (3) and then back down again; (4) and then back up. Like it’s ever lasting”.

Ezra and Jude also described how the water moved up the pipe, then flowed back into the same system, and using a series of gestures, they depicted this (Figures 12 and 13):

Ezra used gesture to add more detail about the motion of the water inside the tube (Figure 14):

**Figure 15.**
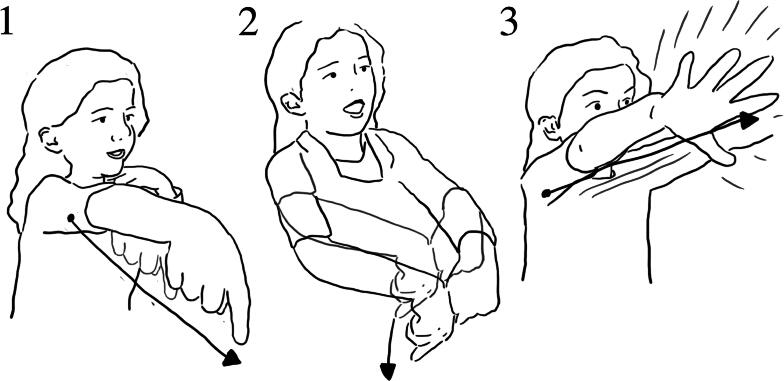
(1) One was going far away down (*draws a point with her finger, in the air, far away from her body*); (2) one was just going down (*brings two index fingers down close to her own body*); (3) one was straight (*moves both palms outwards at fast speed away from her body at chest level*).

**Figure 16.**
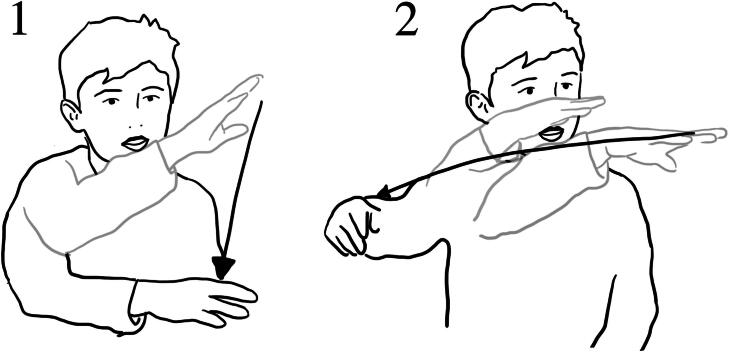
“Some of them were steep” (*gesturing “steep” upwards)*; (2) “and some of them were like that” *(gestures horizontally)* “and then going down” *(gesturing downwards motion).*

**Figure 17.**
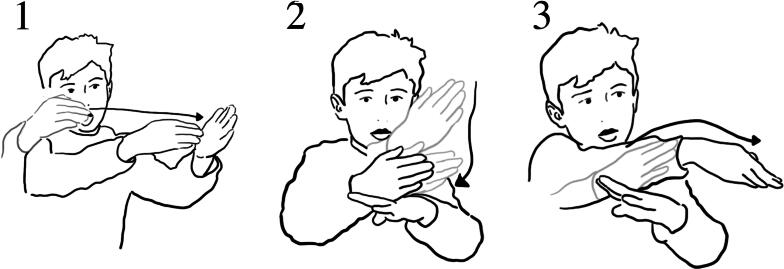
(1) If you did it like that *(uses his left hand to represent the wall of the tub and his right hand to represent the water “pushing” on that surface*) it would stay there … because they’re [the beads] small (3) but if you did it like this because the leaks are there, it might push off.

Through his description Ezra demonstrated an understanding of the mechanisms of the screw. His gestures demonstrate the making of links between his own actions, the shape of the spiraling tube and the direction of the water moving. He gave a detailed and systematic description of how, with each rotating hand motion, more water entered the tube and moved existing water higher up. While his verbal reflection on his interaction experience was broad, his gestures illustrated the degree of detail he had observed.

These examples suggest that the design supported a direct linking between action and effect. This link was enabled both through the transparency of the object itself (the water moving through the screw is visible) and through the repetition of a specific action (rotating) that linked directly to the movement of water, observable close-up over time. We suggest this is a form of embodied proximity, which enabled the children to make explicit links between their own actions and how they effected the system and in so doing supported developing understanding of the mechanism of the Archimedes screw.

#### Water tub

With the water tub children identified differences in water flow in terms of speed, direction, and relative trajectories. They also made connections between the position of the hole relative to the level of the water and the water flow. In the post interaction interview around the water tub, Anna used gesture to depict how the water flowed from the holes in the tub with different trajectories. She described these as “far away down”, “a bit down” and “straight” (Figure 15):

Anna’s gestural expression simulates the speed, direction, and trajectory of different water flows from the holes in the tub. The simple comparative design enabled Anna to explore the different water trajectories, both through feeling (tactile sense) as well as seeing the water flow. In this way, the object fostered a form of embodied proximity.

Similarly, Ezra used gesture to depict the differences in trajectories of water, but also suggested that the height of the hole and “gravity” influence the trajectory of flow (Figure 16).

During interaction, Ezra and Jude also discovered that the small plastic beads could block the holes when being held in position by the water pressure inside the tub. They continued exploring this phenomenon by carefully positioning the beads alongside the holes, both inside and outside the tub. In the interview they talked about the holes “sucking things in”, or if placed on the outside they “just fell off”. Ezra’s gesture communicated how the bead was “sucked” onto the inside of the tub, but pushed off the outside:

Ezra represents the wall of the tub with his left hand and his right hand represents the water flow. Here he positions his right hand on the other side of the tub to show that as the water flows through the holes of the tub there is no longer a surface for the balls to be trapped against, so they “push off” (Figure 17). Through his explanation Ezra demonstrated his developing understanding of how water pressure and flow was needed to hold the ball in place. His gestural expression was linked to his interaction experience, supporting and extending his communication.

These findings suggest that children observed and explored the different water trajectories at different hole heights and water flow dynamics through tactility and vision. Children engaged in direct multisensory experiencing with science ideas through blocking and releasing holes with their fingers, observing how their actions caused the water flow to begin or stop. The design afforded comparison between differences in water flow which was accessible to children as they spent time exploring these differences. In this way, the object afforded embodied proximity, enabling access to science ideas through sensorimotor interaction.

#### Water wheel

**Figure 18.**
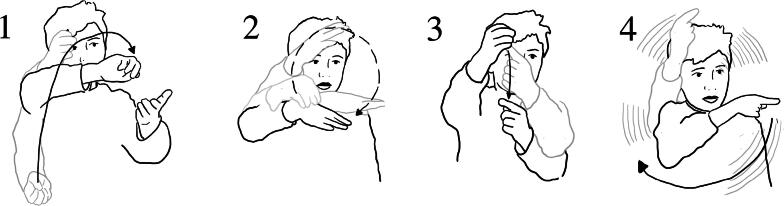
(1) “when I filled it up and then poured it on, it went like that” (*pouring action*); (2) (*reenacts a slow spin of the wheel*); (3) “but when I just did it with the hole” *(forms one hand as if grasping cup and gestures to where the hole in the cup would be);* (4) “it went like that” *(reenacts a fast spin of the wheel).*

**Figure 19.**
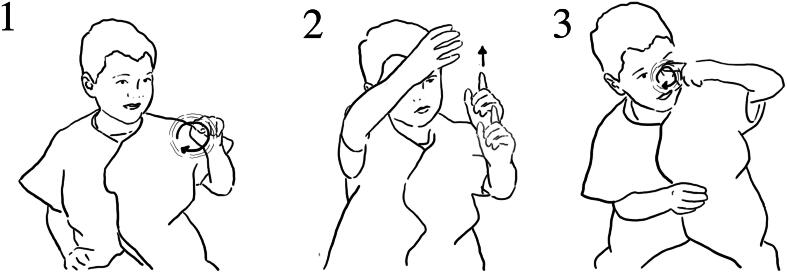
(1) it (*the water*) moved like quick, *gesturing with finger in a circular movement;* (2) cause when we got the water in the cup, *reenacts filling the cup and holding it up high;* (3) the water came out and it hit the spoons and it went round and round like quick, *gesturing with finger in a very fast circular movement.*

Children were able to make connections between the position and rate of the water onto the wheel, the continuity of flow and the speed of spinning of the wheels. The particular actions of children’s hands over the wheel helped to draw attention to the position and volume of water flow and the spinning motion of the wheels (Figure 18):

Here Ezra makes a link between the kind of water flow—short, large volume versus continuous targeted water flow onto the wheel—and the spin speed of the wheel. Similarly, Sam demonstrated how the water wheel moved when they allowed water to flow from the cup onto the wheel (Figure 19).

Some children made links between the size of the blades and the speed of rotation. However, whilst children considered that this variable was important, it was not always evident to them that the wheel with the larger spoons span faster. Some children thought the wheel with the smaller spoons span faster because they were “lighter”, others thought the wheel with the bigger spoons span slower because it was “heavier”. The spin speed was relatively similar and fast at its peak, which made differences hard to observe, and may have been further complicated by the amount and rate of poured water, and typically observing the wheels sequentially, thus having to make comparisons across time. For this object, effects of action were primarily visual, rather than direct multisensorial access; the children could not *feel* the wheel spinning through the tactile sense as the interaction unfolded. This object was perhaps less successful in supporting children’s embodied proximity to science.

## Discussion

**Figure 20.**
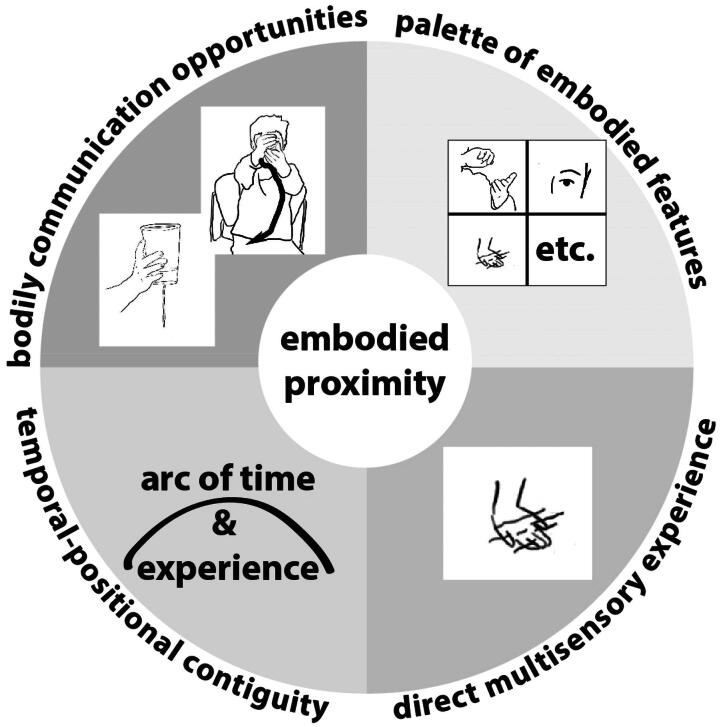
Diagram illustrating key design dimensions for embodied proximity.

While hands-on exhibits for children are mainstream in museums, our research points to the importance of attending to a sensory ‘closeness’ with a science phenomenon in terms of ‘embodied proximity’ or ‘embodied dislocation’. This notion frames a design process that places value on supporting sensorial bodily engagement, in line with theories of embodied learning. The notions of what senses and actions can be interacted with (*sensorimotor features),* and *how* one can directly access some idea through action (*direct multisensorial experience),* as well as the more nuanced details of proximity in terms of body positioning and time *(temporal-positional contiguity)* are all dimensions related to supporting embodied proximity. Study 2 findings point to four key design dimensions for supporting children’s access to and making meaning about science through the body: *considering a palette of embodied features*; *applying direct multisensorial experience*; *developing temporal-positional contiguity;* and *designing for opportunities to communicate details of experience through the body* (Figure 20).

Here, each dimension is discussed within this notion of embodied proximity. Considering the ‘palette of embodied features’ at the early stages of exhibit design aims to identify *how* a child may *use their bodily senses* to access a science idea. Identifying which bodily modalities *can* engage with a phenomenon and *in which ways* can support designers in adopting an embodiment perspective. This relates to the way in which a child’s action and the effect of this action is made perceivable and salient to them. This may be through how the exhibit affords certain kinds of action, and foregrounds the connection between kinesthetic, tactile, aural, and visual senses, and the science idea.

Adopting *direct multisensory experience* as a design dimension offers designers a perspective that emphasizes what actions, and thus, experiences, are directly aligned with a science idea. With the Water Tub, for example, children had access to explore changes in water flow directly through tactile interaction with the holes on the side of the tub. The interview revealed that children had experienced the water moving into several different positions (Anna) in detailed ways (Ezra). This contrasts with Lucy in Study 1, where she reflected on her rotating of a pump, but did not notice the effect on the water—her actions created an effect but this was not noticed since the visual feedback was somewhat far away, and there was no direct way, such as through the tactile sense, to explore those changes.

The dimension of *temporal-positional contiguity* was shown to help understand how, for example, repetitive actions with objects such as Archimedes’ screw afforded an experience that unfolds in time. Or, for example, holding a receptacle that enables water to fall in a continuous manner, affording temporal exploration of the wheels’ spinning. These objects also afforded different kinds of body positional contiguity (or closeness) in interaction; the Screw supported *close-up observation* of water, while the Water Tub afforded positional contiguity through direct active engagement with water and different holes through several iterations. The *wheel* afforded more static positional contiguity, enabling a child to stand next to the wheel holding up a cup of water still and observe.

We argue that *designing for bodily communication* can also foster embodied proximity. This dimension emphasizes the potential for actions in interactive events affording fruitful ground for reflecting and communicating through detailed gestural and bodily language. Our study showed how experiences that foregrounded embodied proximity during an interaction supported children expressing science ideas. Their embodied experience was observed to underpin their communication of ideas through speech, gesture, and reenactment, substantiating previous work (Thomas et al., 2021). It may be beneficial for museums to consider how embodied forms of communication can be supported and encouraged on the museum floor. This dimension, thus, extends existing social embodied learning design frameworks that foreground social interaction (e.g., Danish et al., 2020) by highlighting the importance of also considering embodied dialogue as part of design. Considering that gestural communication forms an important part of children’s communication, especially for younger children whose verbal skills are less sophisticated, spaces that invite children and family members to reflect on their experience, through gestural and bodily communication would enhance embodied proximity to science experience.

## Conclusion

Exploiting young children’s bodily interaction in designing science exhibits is important for fostering young children’s museum-based learning and engagement. Focusing on young children’s interaction with water table exhibits, this qualitative research makes a contribution to exhibit design by providing insight into the nuances of children’s bodily and multisensory interaction with science ideas. Taking an embodied learning approach, the first study revealed barriers to children’s interactions successfully unveiling science ideas. Analysis pointed to the importance of designs that foster embodied proximity, rather than embodied dislocation. The second study examined children’s interaction with, and science reflection around, purpose-built objects based on three design dimensions that foster embodied proximity by attending to: sensorimotor opportunities, direct sensorial experience and temporal-positional contiguity. Findings revealed details of how children’s embodied experience with science-themed objects supported their science thinking pointing to how the notion of embodied proximity revealed the kinds of interaction and engagement the objects afforded, and the level of detail and depth children later communicated about their science idea. The four dimensions (Figure 20) which emerged - *considering palette of embodied features*; *applying direct multisensorial experience*; *developing temporal-positional contiguity;* and *designing for opportunities to communicate details of experience through the body* - provide guidelines for designing museum exhibits that support children’s access to and making meaning about science through the body.

A limitation of this work arises through interaction with the objects in study 2 being separately examined rather than as an integral part of a larger exhibit, as explored in Study 1. Future work could address this by integrating similar objects into a larger water exhibit on the museum floor.

## Figures and Tables

**Table 1.** Design considerations for ‘Archimedes screw’ for supporting children’s embodied proximity to science ideas.

Archimedes’ screw		
Sensorimotor interactive features	Direct sensory experience	Temporal-positional contiguity
Turning handle at the base of the tube enables manual instigation of water movement (tactile/action). Transparent tube through which water flows (vision). Food dye draws attention to position of water in tube (vision).	Direct link between action and water moving up/ down the tube. Mapping action to effect: Turning the handle in one direction causes water to move up the tube. Turning the handle in the other direction causes water to go down the tube (or nothing to happen if tube is empty)	Action has immediate and proximal effect on water movement that can be continually perceived through vision. Direct connection between rotating the handle, and water ‘traveling’ up the tube until it reaches the top and falls into the tray.

**Table 2.** Design considerations for water wheels for supporting children’s embodied proximity to science ideas.

Water wheels		
Sensorimotor interactive features	Direct sensory experience	Temporal-positional contiguity
Holding a cup with a hole at its base in the air (action) enables control of water flow rate, observation of water falling out in a stream and moving the wheel’s blades in a continuous fashion until water is finished (vision/aural). Emptying a cup of water onto the wheel (action) enables motor sensation and observation of water hitting the wheel’s blades making it spin (vision). Observing the different speeds of the two wheels which have differently sized blades (vision).	Action-effect foregrounds visual (and possibly aural) experience of water spinning the wheel. Tactile interaction plays an important role in ‘launching’ the cause-effect chain.	Pouring water onto the blades makes the wheels spin directly. Action has immediate and proximal effect on water wheel movement, or not – if the water flow does not hit the blades correctly. Continuous pouring afforded by the cup with a hole enables perceiving of water flowing onto the blades at a consistent rate over time.

**Table 3.** Design considerations for water tub for supporting children’s embodied proximity to science ideas.

Water Tub		
Sensorimotor interactive features	Direct sensory experience	Temporal/positional contiguity
Fill tub with water (action), observe beads floating and water flowing from holes (vision). Observe beads stopping water flow when they arrive at a hole on the side of the tub, blocking the flow (vision). Stop water flow by pressing on a hole on the side of the tub with one’s fingertip (vision/tactile).	Direct link in pressing and removing one’s finger from holes on the side of the tub to make, and experience, through one’s own action, changes to water flow. Feel water flowing out of holes (tactile) and feel/observe differing trajectories of water depending on position of the hole (top, middle, lower), amount of water above the hole, how many holes are open (tactile/vision).	Action has immediate and proximal effect on water trajectory and water flow, this can be explored on an ongoing basis. Notice volume of water and flow rate changes as it drains; Volume empties slowly giving opportunity to notice changes over time.
